# Influence of aflatoxin B1 on copy number variants in human leukocytes in vitro

**DOI:** 10.1186/s13039-015-0131-x

**Published:** 2015-04-09

**Authors:** Tigran Harutyunyan, Galina Hovhannisyan, Nelly Babayan, Moneeb AK Othman, Thomas Liehr, Rouben Aroutiounian

**Affiliations:** Department of Genetics and Cytology, Yerevan State University, 1 Alex Manoogian, 0025 Yerevan, Armenia; Institute of Molecular Biology, National Academy of Sciences, 7 Hasratyan, 0014 Yerevan, Armenia; Institute of Human Genetics, Jena University Hospital, Friedrich Schiller University, Kollegiengasse 10, Jena, D-07743 Germany

**Keywords:** Aflatoxin B1, Mycotoxins, Copy number variation, Parental origin determination fluorescence *in situ* hybridization (pod-FISH)

## Abstract

**Background:**

Aflatoxin B1 (AFB1) is a mycotoxin produced by *Aspergillus spec.* The latter are worldwide contaminants of food with mutagenic and carcinogenic activities in animals and humans. AFB1 was shown to have deleterious effects on metabolism of eukaryotes in many model systems, including the ability to inhibit DNA replication. An agent that disturbs DNA replication may also have the potential to induce de novo DNA copy number variations (CNVs).

**Results:**

Blood samples of three clinically healthy carriers were treated in vitro with AFB1 and chromosome preparations were subjected to parental origin determination fluorescence *in situ* hybridization (pod-FISH). Probes able to visualize CNVs in 8p21.2 and 15q11.2 were applied. In this setting here for the first time an influence of AFB1 on molecular-cytogenetically detectable CNVs could be shown.

**Conclusions:**

The obtained results indicate that: (i) pod-FISH is a single cell directed, sensitive and suitable method for the analysis of mutagen induced CNVs, (ii) AFB1 has the potential to induce *in vitro* instability of known CNVs in human leukocytes.

## Background

Aflatoxins are mycotoxins produced by *Aspergillus flavus* and *Aspergillus parasiticus*, which are common food contaminants [1,2]. The most important aflatoxin in terms of toxic potency and occurrence is aflatoxin B1 (AFB1), which is also considered as strong carcinogen [3,4]. Studies on the mutagenic effects of AFB1 have shown that it induces chromosomal aberrations in human cells [5-7]. Besides it also acts on DNA level: AFB1 treatment e.g. causes transversion of G/T in 249 codon of the *TP53* gene in human hepatocytes [8]. However, mutational spectrum induced by AFB1 requires further investigation.

DNA copy number variations (CNVs) are defined as stretches of DNA segments ranging in size from one kilobase pair to several megabase pairs when studying different individuals and/or different tissues of an individual. CNVs may occur both in clinically normal and affected subjects [9,10]. Up to 12% of genome is constituted by CNVs, which can arise both meiotically and mitotically [11,12]. CNVs in the normal population, have recently gained considerable interest as a source of genetic diversity. At the same time it is clear that many CNVs have deleterious consequences. Spontaneous or de novo CNVs are an important cause of genetic and developmental disorders, and they also arise frequently in cancer cells [13-15]. Despite their huge impact on human polymorphism and diseases, still little is known about environmental factors which may induce de novo CNVs. Recently the involvement of replication stress inducers (aphidicolin, hydroxyurea, low-dose ionizing radiation) in CNVs formation was shown [16-18]. The ability of different mycotoxins, including AFB1, to inhibit DNA synthesis in mammalian cells was revealed earlier [19-23], but their possible implication in CNVs formation was not yet studied in detail.

Here we describe the influence of AFB1 on earlier reported cytogenetically visible CNVs of 8p21.2 and 15q11.2 [24,25] in human peripheral blood leukocytes using CNV-specific bacterial artificial chromosomes (BACs) as probes for parental origin determination fluorescence *in situ* hybridization (pod-FISH) [26].

## Results

Human peripheral blood lymphocytes of three clinically healthy individuals were used for analysis of influence of AFB1 on CNVs in chromosomal regions 8p21.2 and 15q11.2 using the pod-FISH approach [26]. Fluorescence intensities of signals reflecting the sizes of the CNVs were compared between homologous chromosomes in each metaphase as well as between treated and untreated samples (see below in Methods part “Statistical analysis”).

**Figure 1:**
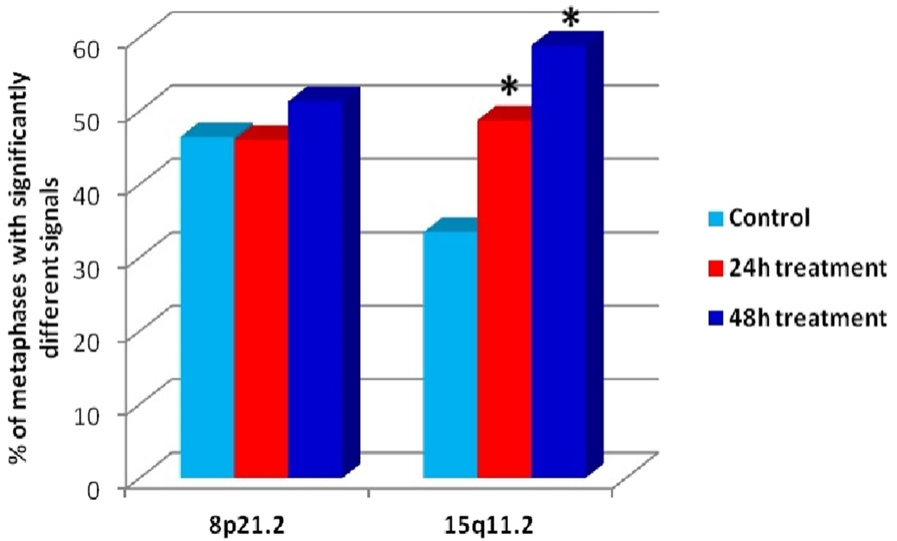
**Percentage of metaphases with significantly different signals in 8p21.2 and 15q11.2 in AFB1-treated human leukocytes.** Fluorescence intensities of signals in chromosome regions 8p21.2 and 15q11.2 were measured in 150 metaphases for each loci by Scion Image program. AFB1 induced statistically significant increase in metaphases with significant differences in CNVs signals between homologous chromosomes in region 15q11.2 in comparison with control (Chi-square test, *p < 0.05).

Evaluation of differences in CNVs signals intensities between homologous chromosomes in each metaphase spread by Chi-square test (Figure 1) indicated for the ability of AFB1 to affect the analysed chromosomal regions. Statistically significant (p < 0.05) increase in percentage of metaphases with significantly different signals in region 15q11.2 (48.75% and 58.9% after 24 and 48 hours of AFB1 treatment, respectively) compared with control (33.45%) was shown (Figure 1). In region 8p21.2 we found only a statistic trend toward instability after 48 hours of AFB1 treatment (51.3%).

**Figure 2:**
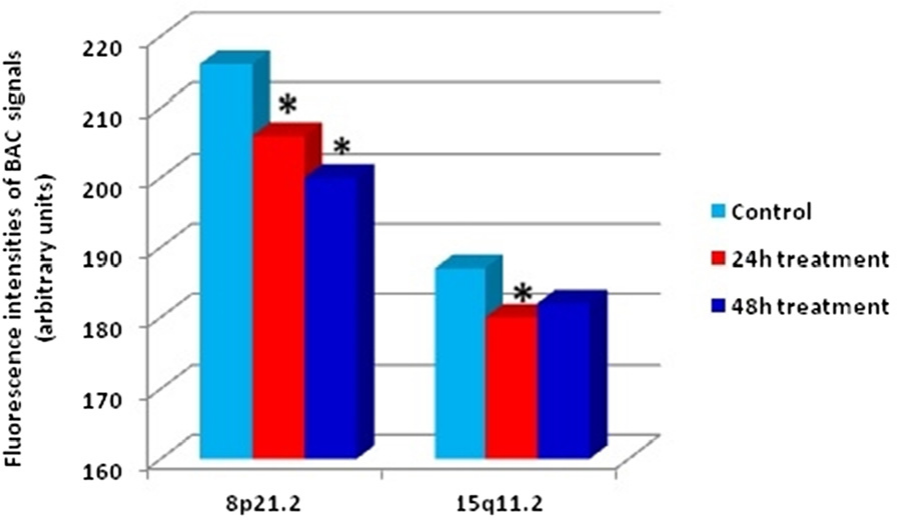
**Fluorescence intensities of signals in chromosome regions 8p21.2 and 15q11.2 in AFB1-treated human leukocytes.** Fluorescence intensities in chromosome regions 8p21.2 and 15q11.2 were measured in 150 metaphases for each loci by Scion Image program. AFB1 decreased mean values of fluorescence intensities of signals in regions 8p21.2 and 15q11.2 in comparison with controls (Mann-Whitney W-test, *p < 0.05).

Kolmogorov-Smirnov test revealed that the distribution of signals intensities of chromosome regions 8p21.2 and 15q11.2 is non-normal, which precludes the use of parametric tests. Thus, nonparametric Mann-Whitney W-test was applied for comparison of CNVs between AFB1-treated and untreated cells (Figure 2). Analysis of obtained results revealed that AFB1 induced decrease in signals intensities in the selected chromosome regions compared to control. Namely, the levels of CNVs were significantly (p < 0.05) decreased in region 8p21.2 after 24 (206 a. u.) and 48 hours (200 a. u.) and in 15q11.2 after 24 hours (180 a. u.) of AFB1 treatment in comparison with controls (216 a. u. and 187 a. u., respectively). Tendency to decrease was also observed in 15q11.2 of AFB1-treated cells after 48 hours (182 a. u.).

Overall, we provide the first evidence of AFB1-induced instability in two CNV loci of human genome. Decrease of the size of CNV loci permitted to suggest that the instability might occur mainly due to deletions in the studied regions.

## Discussion

There are many publications related to analysis of spontaneous CNVs in human population [9,13,15]. However, little is known about the induction of de novo CNVs by environmental risk factors [16].

The main sources of CNVs are duplications and deletions, and there are different models that explain molecular mechanisms of these processes. Change in copy number involves change in the structure of the chromosomes which occur by two general mechanisms, nonhomologous end joining, along with homologous recombination [11]. Both of these mechanisms are involved in repair of arrested replication forks that include a double strand break [27,28]. Different studies have shown that inhibitors of replication may induce CNVs experimentally in human cells [17,29]. Particularly, the DNA polymerase inhibitor aphidicolin and replication inhibitor, hydroxyurea were correlated positively with increased induction of CNVs incidence in somatic cells cultured in vitro [16].

Earlier it was shown that AFB1 is mutagenic in many model systems and produces chromosomal aberrations, micronuclei, sister chromatid exchange, unscheduled DNA synthesis, and DNA strand breaks, as well as forms adducts in rodent and human cells [30]. There is evidence that the predominant AFB1–DNA adduct AFB1–N 7-Gua act as replication blocks [31,32]. Based on this information, the purpose of this study was to determine the ability of AFB1 influence on CNVs level in human blood cells in vitro.

The data of our study show that AFB1 impacts CNVs located at chromosome loci 8p21.2 and 15q11.2 in human peripheral blood leukocytes. Significant increase of differences in the sizes of the CNVs between homologous chromosomes was revealed in AFB1-treated cells compared with control. The fluorescence intensities of signals in blood leukocytes decreased after AFB1 treatment in the most cases, indicating deletions in 8p21.2 and 15q11.2. Earlier in AFB1-exposed hepatocellular carcinoma’s cases homozygous deletions at different loci were reported [33] including chromosome regions 8p23 and 15q25-26 adjacent to the areas studied in our work [34-36].

CNVs analysis was based on a comparison of fluorescence intensities in 8p21.2 and 15q11.2 between homologous chromosomes as well as between AFB1-treated and untreated samples. This approach permits to detect unequal loss or gain and does not allow recognizing deletions or duplications of similar size occurred simultaneously in the compared loci. Thus, our results can be considered as underestimated but even so, they do indicate the effect of AFB1 on CNVs.

Taking into consideration the fact that AFB1 is a worldwide contaminant of food its effect on the CNVs in human genome can be quite substantial. Further studies of AFB1-promoting copy number change are warranted to shed light on de novo induced CNVs formation.

## Conclusions

In conclusion, our preliminary results indicate that AFB1 can induce instability in CNV regions in chromosome loci 8p21.2 and 15q11.2 in human leukocytes culture. It was revealed that instability is a consequence of deletions in analyzed regions. This first study on influence of AFB1 on CNVs in human blood leukocytes requires further systematic trials in future.

## Methods

### Blood cultivation and treatment with aflatoxin B1

Blood samples were collected from three healthy volunteers – two female and one male aged 24-26 years. The study was approved by the Ethics Committee of the Institute of Molecular Biology of National Academy of Sciences of RA (IRB # IORG 0002437), and informed consent was obtained from all study donors. The venous blood (5 ml from each donor) was collected into syringe with heparin (0.5 ml) and incubated in 50 ml of RPMI-1640 medium, containing 10% foetal bovine serum, 1% penicillin/streptomycin, and 10 μg/ml phytohemagglutinin-L at 37°C. The cells were treated with AFB1 (Sigma - A6636) dissolved in 96% ethanol 24 and 48 hours after cultures initiation. The final concentration of AFB1 in the cultures was 3 μg/ml. Due to the limited publications on de novo induced CNVs the AFB1-treating model was developed based on the data on chromosomal aberrations [5-7,37] and personal experimental results.

### Metaphase chromosome preparation

Metaphase chromosomes were prepared according to Bangs and Donlon [38]. Colcemid (0.1 μg/ml final concentration) was added to the culture 2 hours before harvesting and incubated at 37°C to achieve metaphase block. In total, blood cultures were incubated for 72 hours at 37°C. At the end of cultivation cells were harvested and centrifuged at 1500 rpm (10 min). The medium was removed completely except for about 0.5 ml of supernatant remaining above the cell pellet. 10 ml of pre-warmed (37°C) hypotonic solution (0.075 M KCl) was added to the tubes and the contents were mixed gently and incubated for 15 minutes at 37°C. Then a few drops of freshly prepared fixative (methanol/glacial acetic acid, 3:1) were added and inverted to mix. After centrifugation and discarding supernatant cells were fixed by 10 ml of ice-cold fixative. After incubation 10-15 minutes at room temperature the cells were centrifuged, supernatant was discarded and 10 ml of fixative was added. After the last centrifugation, cells were resuspended in a small amount of fixative and the suspension was dropped onto a microscope slide, prewashed by fixative. Then the slide was placed on hotplate (51°C) covered by wet tissue paper and kept until the surface of the slide was dried.

**Figure 3:**
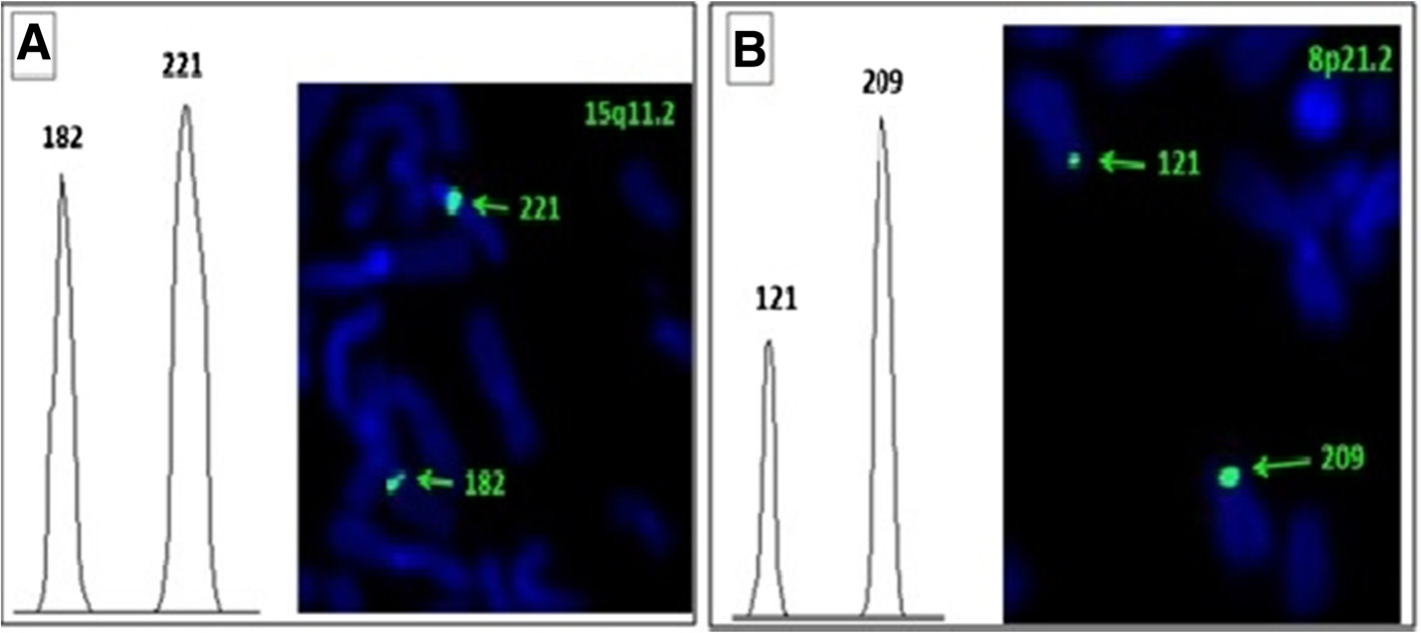
**Sample of evaluation of signal intensities by Scion Image program (highlighted by arrows).** Signals intensities measurements were done on homologous chromosomes of 15q11.2 **(A)** and 8p21.2 **(B)** after incubation with AFB1 in a. u. CNV-specific BAC probes RP11- 79C23 for chromosome region 15q11.2 and RP11-115K10 for chromosome region 8p21.2 were applied.

Parental origin determination fluorescence *in situ* hybridization (pod-FISH) has already been successfully used to identify CNVs in human cells [39]. BAC clones from CNV regions were selected by the “Database of Genomic Variants” (http://dgv.tcag.ca/dgv/app/home), purchased from the Children’s Hospital Oakland Research Institute (CHORI), Oakland, CA, USA, or kindly provided by the Sanger Centre, UK. BAC DNA was isolated, PCR amplified, and labeled by nick translation (Roche, Karlsruhe, Germany) [26]. The following BACs were used: RP11-115K10 and RP11-79C23 for 8p21.2 (SpectrumGreen) and 15q11.2 (SpectrumGreen) regions, respectively. Image capturing and acquisition were processed with the Isis imaging system (MetaSystems, GmbH, Altlussheim, Germany). For analysis of pod-FISH signals the freeware SCION (http://scion-image.software.informer.com/) (Figure 3) was applied [40]. For that purpose images were converted to grayscale and fluorescence intensities of signals were measured from 150 metaphases for each region and expressed in arbitrary units (a. u.).

### Statistical analysis

The normality of distribution of FISH signals intensity was analysed by the Kolmogorov-Smirnov test (was confirmed by analysis of standardized skewness and standardized kurtosis). Chi-square test was applied to analyze the significance of difference of homologous chromosomes signals. First, in every metaphase the signal intensities of homologous chromosomes were compared with each other separately. Second, the obtained quantity of significantly different measurements of signals for each variant in percents was compared to estimate the influence of AFB1 on CNVs. Mann-Whitney W-test (nonparametric test) was applied for determination of difference between treated and untreated groups.

Chi-square test was performed using online interactive calculator [41]. Mann-Whitney W-test was performed using the statistical package Statgraphics 16.2. A probability level at p < 0.05 was considered as statistically significant.
